# Supplementary material for: Mapping global floods with 10 years of satellite radar data
Source: Nat Commun. 2025 Jul 1;16:5762. doi: 10.1038/s41467-025-60973-1 (PMC12216028; doi:10.1038/s41467-025-60973-1)
Supplement: Supplementary file 1 — Supplementary Information [file 41467_2025_60973_MOESM1_ESM.pdf]

Supplementary Information for Mapping Global Floods with 10 Years of Satellite Radar Data

Amit Misra, Kevin White, William Straka, Simone Fobi Nsutezo, Juan Lavista

S1 Neural Network Model

S1.1 Training Data

This subsection details our approach to creating a robust training dataset, including the selection of flood events, the validation process, and the creation of negative samples. Our training data consists of manually labeled SAR images from known flood events. We selected four events from recent years. These four events were selected to represent a diverse range of geographical and climatic conditions, spanning three continents and distinct climate types like arid regions in Ethiopia and Pakistan, tropical savannah in Mozambique, and Mediterranean in Greece.

- Pakistan flooding in August 2022
- Greece flooding in September 2023
- Mozambique flooding in March 2023 (validation scene)
- Southeast Ethiopia flooding in November 2023 (test scene)

Despite the availability of several public datasets for flood detection using satellite data, we opted to create our own hand-labeled dataset for two main reasons: we wanted to focus on change detection, which requires a temporal aspect to the data (essentially a pre image and post image), and we wanted to use SAR data only. Our initial analyses suggested the importance of a change detection approach, and at the time there were no datasets that included SAR data with both pre and post flood imagery. Additionally, we wanted to focus on SAR data, and many previous data sources where either based on optical imagery (or optical and near-infrared) or used a combination of SAR and optical. This led to cases where flooding would be detected in optical imagery but not in SAR. While these are true detection of flooding it can make it more difficult for the models to learn to identify flooding. Therefore, we elected to only consider cases where we could identify flooding in SAR data. (Note: since the preparation of this manuscript, an additional dataset for flood detection using SAR data has been released. We leave analysis of this dataset for future work.)

We selected these events based on clear evidence of flooding in the SAR data, and we can validate that the flooding is not a false positive from other sources, such as Sentinel-2 optical imagery, news articles, drone footage, or UN reports. Because there are potential false positives, it's difficult to look just at a SAR image and confidently label anything as flooding without external data included. We explore false positives in more detail in Section S2.

An example of our dataset validation process is shown in Supplementary Figure S1, which displays SAR imagery from the Greece floods in September 2023. We can clearly see the change in SAR backscatter between August 26 and September 7. This flood was widely covered in the news, with specific reports of impacted locations. By correlating publicly reported flood impacts with areas showing changes in SAR imagery, we can increase our confidence that observed detections represent true flooding. While we cannot verify every pixel individually, this type of cross-referencing gives us greater confidence in the overall accuracy of the labels.

For negative samples, we carefully select areas within the same scenes as the flood events where flooding is not evident in the SAR imagery. We focus on regions that could potentially be misclassified as flooded, such as areas with isolated pixels showing flood-like signals surrounded by non-flood pixels. We then cross-reference these areas with the other datasets mentioned above to confirm they were unlikely to have been flooded, and label them as non-flooded. This approach ensures our negative samples include challenging cases that help refine the model's ability to distinguish between truly flooded areas and similar-looking non-flooded regions. When available, we also confirm with other sources, such as cloud-free Sentinel-2 imagery.

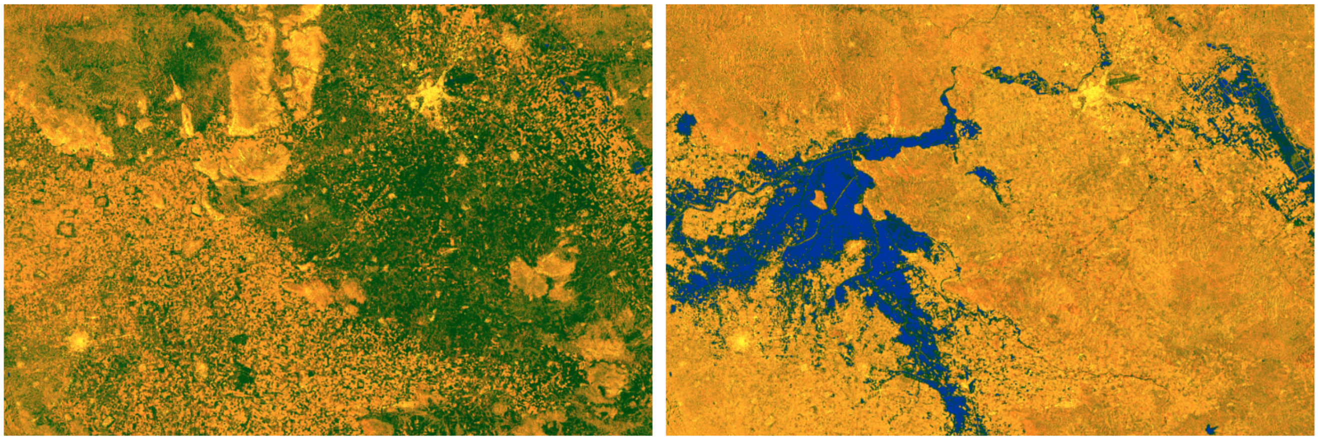

Supplementary Figure S1: False-color SAR images before (a) and after (b) the flooding in Greece in September 2023. The flooding is clearly visible as the dark blue in the west of the post image. SAR images were retrieved from Microsoft's Planetary Computer.

S1.2 Model Architecture

For this task, we employed a MobileNet early fusion change detection model. Specifically, we used pairs of SAR images, matched for time of day and geographic bounds, taken within 30 days of each other. In this context, 'early fusion' refers to the process of combining spatial and temporal information early in the processing pipeline. Specifically, our model takes two SAR images as input - one before and one after a potential flood event - and merges their features in the initial layers of the network. This approach allows the model to directly learn spatiotemporal patterns indicative of flooding, as opposed to late fusion methods that process each image separately before combining their outputs.

We selected a MobileNet architecture for its lightweight nature, which ensures fast inference times — a crucial factor for real-time flood detection applications and for running inference at a global scale for nearly 10 years of data. Although we tested more complex architectures like ResNet18 and ResNet50, our results indicated comparable performance in terms of precision, recall, and F1 score. This finding suggests that a lightweight model is sufficient for the SAR imagery used in this study, without the need for additional computational overhead associated with more complex models

For each model, we also tested fine-tuning, which involves using a pretrained model (e.g., trained on ImageNet) and then adapting it to our specific task. Ultimately, we opted for a non-fine-tuned MobileNet model due to its superior performance on the validation dataset.

S1.3 Input Features

We initially experimented with using the VV and VH amplitudes (decibel scaled) as direct inputs to the model, but found that the model performance on the validation dataset improved when we explicitly applied some filtering to the SAR amplitudes to identify the ranges of pixel values consistent with the presence of water. We first identified the range of typical water values for our training data, looking at permanent water (rivers, lakes and oceans) in the scenes. Based on the values of the VV and VH backscatter amplitudes in the scenes, we identified minimum and maximum thresholds for water. This method of global thresholding for detecting water is common in analysis of SAR imagery. A drawback of setting global thresholds is that the appropriate thresholds to set may vary based on geographic region. One potential future improvement in the model is to use local thresholding, with thresholds determined for different regions/images and also done dynamically for different land cover types present in the image. Alternatively, with more training data, the model could likely learn the correct relationships purely based on the amplitudes with no filtering applied. We leave this for future work.

We then use these thresholds to determine whether a pixel has transitioned from outside the range of likely water values in the pre-image to within the range of water values in the post-image for each of the SAR polarization bands. We also include features on the difference in amplitude. The intuition behind adding these delta amplitude features is that a

large change in amplitude is more likely to be correlated with true flooding than a small change. If a pixel has an amplitude that is near the threshold of water versus not water, a small change could result in a binary change for that polarization channel. However, those small changes could be due to noise. Including the delta amplitude features allows the model to learn which combination of binary changes and changes in backscatter amplitudes are correlated with true flooding.

This results in four features that are the inputs to the model. We selected this feature set via performance on the validation data set.

- VV binary change (transition from outside to inside the water range)
- VH binary change (transition from outside to inside the water range)
- VV delta amplitude
- VH delta amplitude

S1.4 Model Training

We employed basic image augmentation techniques during model training, including vertical and horizontal flips as well as rotation, to increase the diversity of the training data and improve the model's robustness to variations in the input images. A softmax activation layer was used to produce the final class probabilities.

For hyperparameters, we optimized the learning rate, batch size, and loss function using the validation dataset, with the Intersection Over Union (IOU) metric serving as our primary measure of performance. We used a grid search over parameters to determine the optimal hyperparameters. We evaluated three loss functions: binary cross entropy, dice loss, and focal loss. The final hyperparameters are shown in Supplementary Table S1.

Hyperparameter	Value
Learning Rate	0.001
Batch Size	32
Loss Function	Dice Loss

Supplementary Table S1: Final Hyperparameters used for Model Training

To prevent overfitting and ensure optimal model performance, we implemented early stopping during the training process. Early stopping monitors the validation loss and halts training if the loss does not improve for a specified number of epochs, known as the patience parameter. We set the patience parameter to 8 epochs, meaning training would stop if the validation loss did not decrease for 8 consecutive epochs. The maximum number of epochs was set to 30. In practice, for our model we found that early stopping did not significantly change evaluation metrics on the training or validation datasets, but did reduce training time in the final model training runs.

S1.5 Model Validation

As described in the Methods section, we validated our model using test set metrics and the Kuro Siwo flood dataset. Here we provide additional technical details about our validation approaches and results, along with an evaluation of the model predictions against high-resolution satellite imagery.

S1.5.1 Test Set Validation

While the key metrics are reported in the main text, several additional considerations informed our model development. We tested adding soil moisture and elevation data into the model training and testing, but given the lack of diversity in positive examples with low soil moisture and elevation or slope across the labeled data, we found no improvement in model performance. We therefore elected to include these features in post-processing instead. The high recall (0.99) achieved by our model is particularly desirable given our approach of using the neural network to identify potential flooding candidates, followed by heuristic filtering to remove false positives.

S1.5.2 Kuro Siwo Validation

The Kuro Siwo dataset provides an ideal independent validation source for our model, encompassing 43 historical flood events across 6 continents and multiple climate zones. This global coverage, combined with multi-temporal imagery and both VV/VH polarizations, makes it uniquely suited for comprehensive model validation. However, one key consideration is

that Kuro Siwo uses GRD (Ground Range Detected) SAR data, rather than the RTC (Radiometrically Terrain Corrected) data our model was trained on. While GRD data offers advantages in processing time and flexibility, the lack of terrain correction introduces a potential bias towards worse model performance.

To account for these GRD/RTC differences, we derived new VV and VH thresholds for identifying water using 5 European locations from the Kuro Siwo dataset. We selected Europe as it contains the most flooding scenes, choosing random locations with at least 1% permanent water pixels that were not part of our test dataset. This allowed us to fairly evaluate model performance across different geographies while accounting for data processing differences.

For each scene in the Kuro Siwo dataset, we ran our model twice - once against each pre-flood image - and took the maximum flood prediction from these runs, then applied an 80-meter buffer (see Section S1.5.4 for buffer selection methodology). While this buffer size was selected based on our analysis of high-resolution imagery in Kenya, subsequent experimentation with the Kuro Siwo dataset independently confirmed this as the optimal value.

We evaluated our model against the Copernicus Global Flood Monitoring (GFM) predictions as an additional baseline. GFM provides probabilistic flood likelihood values, offering flexibility in balancing precision and recall through threshold selection. Using the same 5 European locations mentioned above, we optimized GFM's flood likelihood threshold by maximizing F1 score. The optimal threshold of 0.3, determined using only these locations, proved to be the best-performing threshold across the entire Kuro Siwo dataset. This contrasts with our model's binary flood/no-flood predictions, though both approaches showed strong performance in flood detection (see Table S2). To ensure fair comparison between models, we excluded permanent water bodies as defined in the GFM dataset from all analyses, as GFM and Kuro Siwo use different permanent water masks.

While our main text presents high-level comparisons between our model, the Kuro Siwo baseline, and GFM predictions, Table S2 provides a detailed continental breakdown of F1 scores. The table shows results for both baseline and thresholdoptimized versions of each model. For both models, optimizing thresholds generally improves performance, though the effect is more pronounced for GFM. The larger improvement in GFM's performance (e.g., from 0.52 to 0.71 in Africa) reflects how lowering its detection threshold increases recall and improves overall F1 scores. For our model, the more modest improvements from threshold tuning likely reflect adjustments needed to account for differences between the RTC data used in training and the GRD data in Kuro Siwo.

Our model shows particularly strong performance in Asia (F1 = 0.80) and Australia (F1 = 0.76), where it significantly outperforms GFM. Note that European results exclude the locations used for threshold optimization, and we combined North and South America due to limited South American data (one location).

Continent	GFM	GFM (tuned)	AI4G	AI4G (tuned)
Africa	0.52	0.71	0.67	0.75
Americas	0.55	0.75	0.59	0.68
Asia	0.70	0.77	0.75	0.80
Australia	0.34	0.19	0.76	0.76
Europe	0.48	0.64	0.72	0.74
Test	0.63	0.72	0.73	0.77

Supplementary Table S2: Continental comparison of model performance (F1 scores) on the Kuro Siwo validation dataset. GFM and AI4G models are evaluated both with and without threshold tuning. The AI4G model shows strong performance across most continents, particularly outperforming GFM in Australia and Africa. The 'Test' row shows results for the BlackBench test set subset of Kuro Siwo, enabling direct comparison with other published work.

The continental analysis reveals both broad patterns and notable exceptions in model performance. While our model generally outperforms GFM, there are specific locations where performance diverges significantly. We examine three cases that illustrate key aspects of model behavior and data interpretation challenges: flooding in Peru where labeling uncertainty affects evaluation, mudflats in Australia that highlight detection limitations, and flooding in Macedonia where our approach shows particular strengths.

Peru: Label Uncertainty in the Americas. The apparent underperformance of our model in the Americas (F1 score 0.33 vs GFM's 0.76) is primarily driven by a single scene: the March 2017 flooding in Peru. Detailed examination of this case reveals important data interpretation challenges. While our model predicts less flooding than indicated by the Kuro Siwo labels, analysis of Landsat imagery from the same day suggests the actual flood extent was less extensive than the labels indicate,

as shown in Figure S3. This case demonstrates that even high-quality datasets like Kuro Siwo can contain labeling uncertainties that affect performance metrics.

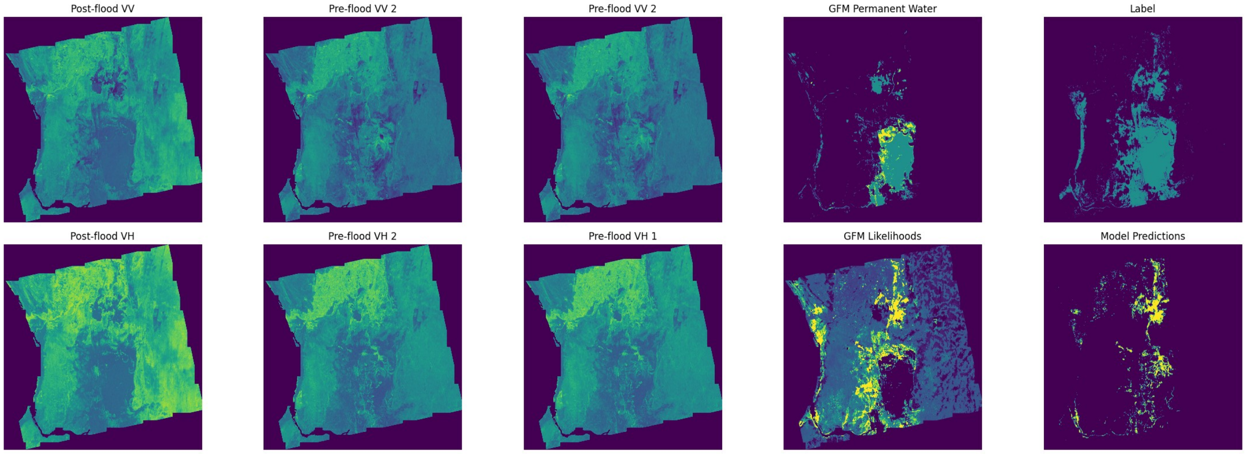

Supplementary Figure S2: Pre-post SAR imagery for VV (top) and VH (bands) along with permanent water from GFM, Kuro Siwo labels, GFM likelihood values and our model predictions for a Kuro Siwo location in Peru. While our model misses much of the flooding that GFM identifies, there is uncertainty in the labels that may be driving our lower model performance.

Australia: Mudflat Detection Challenges. The February 2022 flooding near Burketown, Australia illustrates a key limitation of SAR-based flood detection in mudflat regions. Our F1 score for this scene is 0.42 vs 0.59 for GFM. Figure S4 shows that our model significantly underestimates flood extent in these areas because mudflats appear dark in SAR imagery even under normal conditions. This behavior extends to other 'bare ground' surfaces like desert regions that exhibit consistently low SAR backscatter. While this represents a limitation of our approach, we address it by explicitly marking these areas in our exclusion layer to flag potential detection challenges.

Macedonia: Advantages of Change Detection. The August 2016 flooding in Northern Macedonia demonstrates our model's strengths compared to GFM, achieving F1 scores of 0.71 versus 0.22. As shown in Figure S5, GFM at its optimal threshold (0.3) misses most of the flood extent, while lower thresholds introduce excessive false positives - even its best performance at threshold 0.2 only reaches an F1 score of 0.41. We attribute our model's superior performance to the combination of change detection with deep learning, which better captures flood dynamics for this scene.

Our model's strong performance on the Kuro Siwo dataset, achieving comparable or better results than GFM across most regions, demonstrates its effectiveness as a global flood detection system. The identified limitations, such as reduced accuracy in mudflat regions, primarily affect areas already marked in our exclusion layer, allowing users to appropriately interpret results in these challenging terrains.

It's worth noting that our validation against Kuro Siwo likely represents a conservative estimate of model performance. Two factors contribute to this: first, our model was trained on RTC rather than GRD data, and second, the extended time gaps between Kuro Siwo's pre- and post-flood images introduce seasonal variations in vegetation and soil moisture that our model was not designed to handle. Our model was trained on images less than 30 days apart, while some Kuro Siwo image pairs are several months apart. Despite these challenges, the model's robust performance across diverse global contexts validates its utility for operational flood detection.

S1.5.3 Evaluation against high-resolution imagery

To evaluate our model against independent data sources, we compared our model's outputs with high-resolution imagery from the Kenya floods in April and May 2024, using data from Airbus Pleiades (obtained via Unosat with flood extent provided) and

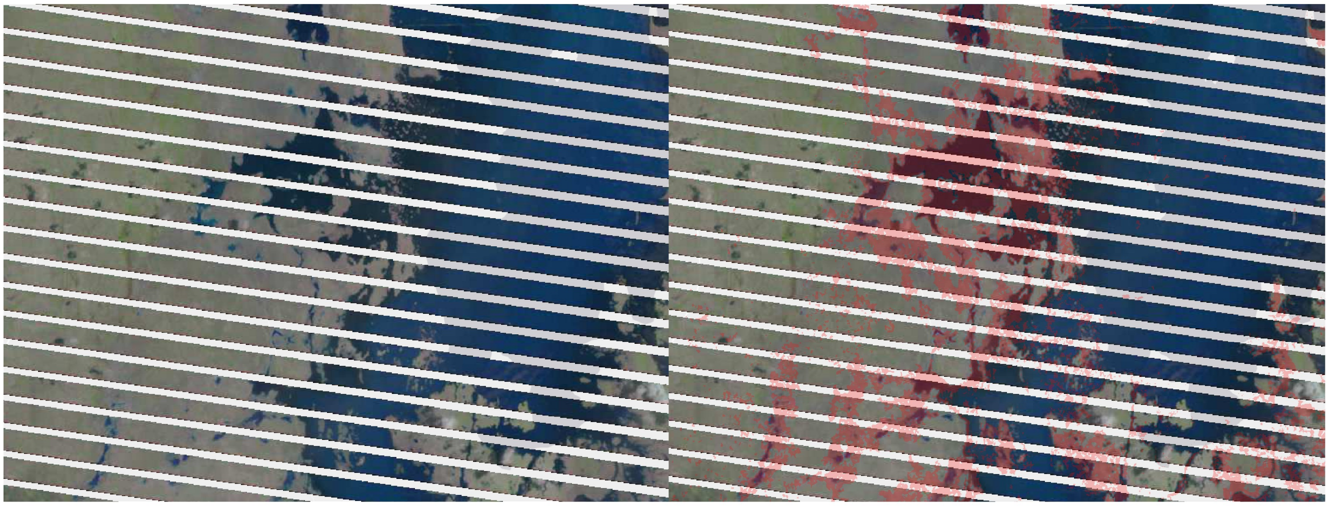

Supplementary Figure S3: (a) Landsat image from the same day as the post image from the Kuro Siwo dataset for part of the scene. (b) Same Landsat image with the Kuro Siwo flood label in red added (permanent water as defined by GFM excluded). There are several areas marked as flooded that do not show any evidence of flooding in the Landsat image. This suggests potential uncertainty in the flood label for this scene that may be driving our model's poor performance in this area. Base map imagery from Landsat 7, courtesy of the U.S. Geological Survey.

Planet Labs across four regions: Nairobi, Busia, Garsen, and Garissa. Additionally, we compared results to the performance of NOAA VIIRS-based flood product and the GFS ensemble flood extent maps.

The model shows considerably higher performance when run on 10 meter input imagery instead of 20 meter imagery (see Table S3). In our standard workflow, we typically use 20 meter resolution data for model inference due to its balance of accuracy and computational efficiency. The 20 meter resolution helps mitigate false positives while maintaining reasonable detail and accuracy for most applications. The choice between resolutions ultimately depends on the specific use case, balancing the need for detail against processing efficiency.

Model	Busia	Garissa	Garsen	Nairobi	Average IOU
AI4G Model - 10m	0.21	0.42	0.45	0.11	0.26
VIIRS	0.20	0.40	0.19	0.06	0.18
AI4G Model - 20m	0.09	0.20	0.22	0.03	0.11
GFS	0.00	0.04	0.15	0.05	0.06
Planet vs Pleiades				0.47	

Supplementary Table S3: Model Evaluation, Kenya, Spring 2024 | Model performance metrics across different locations. Our model (AI4G) generally has good performance, with better performance when higher spatial resolution input imagery is used. Our model results compare favorably against publicly available VIIRS (optical) and GFS (SAR) flood extent estimates.

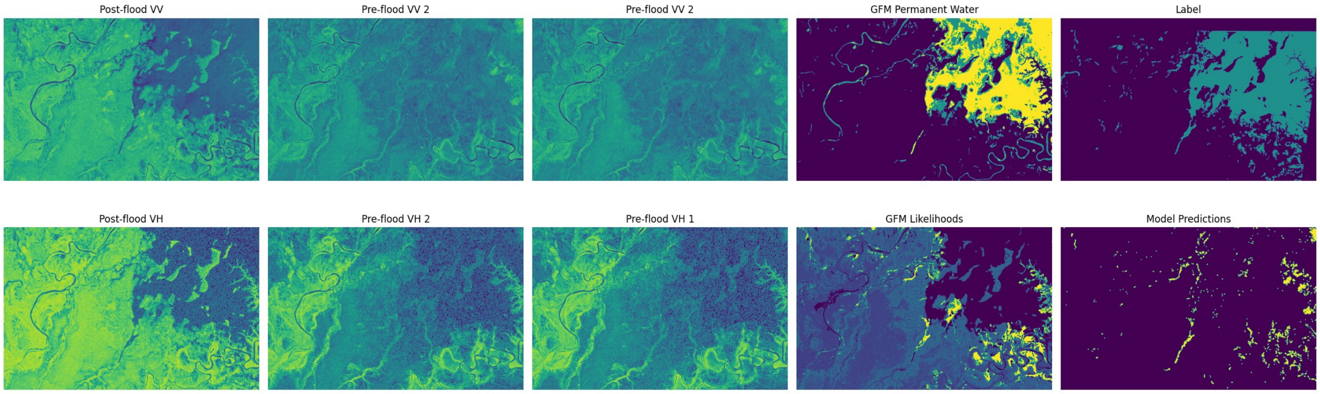

Supplementary Figure S4: Pre-post SAR imagery for VV (top) and VH (bands) along with permanent water from GFM, Kuro Siwo labels, GFM likelihood values and our model predictions for a Kuro Siwo location in Australia. The model has difficulty accurately mapping the flood extent outside the larger mudflat that GFM identifies as semi-permanent water. As seen in the raw VV and VH imagery, much of the flooded area is initially dark in the SAR imagery. Our change detection fails to capture areas like this that already appear in SAR. This specific area, and many such areas, are part of our Exclusion Mask, which allows users to see that flood detections in certain areas are subject to high uncertainty.

The lower performance against high-resolution imagery compared to the test set is primarily due to two factors: noncontemporaneous image timing and differences in flood detection capabilities. While SAR excels at detecting active standing water, high-resolution imagery can also capture flood trace evidence from recently receded waters. As a baseline for comparison, we note that even high-resolution optical imagery from different satellites (Planet and Pleiades) of the same location on the same day showed an IOU of only 0.47, highlighting the inherent challenges in precisely matching flood extent across different observations.

S1.5.4 Selection of optimal buffer

One challenge inherent in high-resolution flood modeling using SAR imagery is that SAR images may not always accurately capture every pixel of a flooded area. Factors such as SAR signal scattering, speckle noise, and surface roughness variations can result in imperfect flood detection, leading to potential underestimation of the flood extent. To mitigate these limitations, we apply a buffering or dilation to the flood detections.

We applied a buffer or dilation around flood detections by treating neighboring pixels to the raw flood detections as positive detections out to a buffer distance. While this removes some of the benefit of having high resolution data, this helps in capturing more of the actual flood extent. We also note that this is a post-processing step which other researchers do not need to use when applying this model or the underlying predictions for their own use cases.

To determine the most effective buffer size, we conducted a series of tests using different buffer values. The results of these tests are in Supplementary Table S4. For Garsen, a buffer of 80 meters results in the best IOU. However, for Busia and Naoribi, increasing the buffer up to 240 meters results in higher IOU, but upon visual inspection that is primarily driven by isolated detections in SAR that when buffered by larger values increased the recall of the model at the expense of precision. We see similar results in Garissa, where the maximum buffer goes out 480 meters. However, we view the 80 meter buffer as more practical and aligned with what the model is detecting as flooding. Therefore, we lean towards an optimal buffer values of 80 meters when trying to estimate the most accurate flood extent.

An additional validation of the choice of 80 meter buffer comes from the Kenyan government's official numbers on cropland affected by the 2024 floods. The government reported that 168,000 acres (roughly 68,000 hectares) of cropland were affected by the floods. Supplementary Table S5 shows the results for estimated cropland affected based on our model results with a buffer included. Our model result with a 80 meter buffer is closest to the official government estimate, providing additional support for a use of a 80 meter buffer for our model.

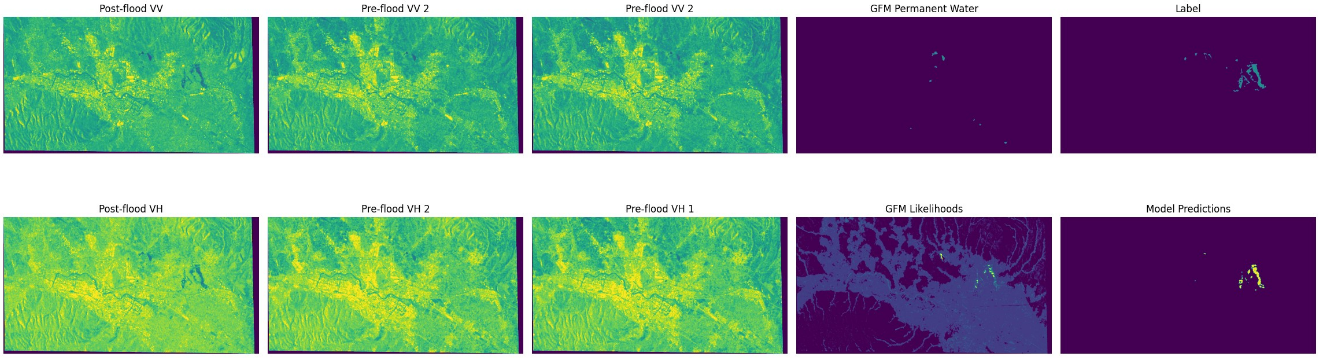

Supplementary Figure S5: Pre-post SAR imagery for VV (top) and VH (bands) along with permanent water from GFM, Kuro Siwo labels, GFM likelihood values and our model predictions for a Kuro Siwo scene in Macedonia. Our model outperforms GFM by a large margin here. While GFM correctly identifies some of the flooding, there are considerable false positives. Even with the optimized thresholds for the GFM likelihoods, there are many locations that are incorrectly tagged as flooded. This is one of the starkest examples where our model outperforms the GFM emsemble predictions.

Buffer Size	Busia	Garissa	Garsen	Nairobi	Avg IOU
20	0.5	0.11	0.20	0.02	0.09
40	0.11	0.23	0.36	0.05	0.19
60	0.16	0.34	0.43	0.08	0.25
80	0.21	0.42	0.45	0.11	0.30
100	0.25	0.49	0.45	0.13	0.33
120	0.28	0.53	0.44	0.14	0.35
240	0.34	0.54	0.28	0.18	0.33
360	0.31	0.54	0.28	0.18	0.33
480	0.26	0.50	0.24	0.17	0.29

Supplementary Table S4: IOU for Busia, Garissa, Garsen, and Nairobi with the average across all four regions.

Overall, 80 meters is a reasonable buffer amount to apply, but in cases where one wanted to take a wider view on risk, 240 meters could be appropriate. For example, if the goal is to identify areas for potential flood mitigation efforts or to create broader flood risk maps for urban planning, a larger buffer could provide a more conservative estimate of at-risk areas. Conversely, for more precise applications such as agricultural impact assessments, the 80 meter buffer provides a balance between accuracy and inclusivity.

Buffer size (meters)	Hectares of Cropland Affected (1000's)
0	8
20	13
40	32
60	53
80	75
100	99
120	123
240	281

Supplementary Table S5: Hectares of cropland affected in Kenya by buffer size. As buffer size increases, more cropland is tagged as affected by flooding. The official government estimate of cropland affected was 168,000 acres, or 68,000 hectares. This is closest to the estimate from our model with a 80 meter buffer applied.

S1.6 Compute

Training and inference were performed on a NC64as_T4_v3 Azure VM with 4 Nvidia Tesla T4 GPUs and 440 Gb of RAM. Model training was conducted on a single GPU and took roughly 8 hours. While model training was relatively quick, the inference process presented significant computational challenges. Model training is done on only 4 flood events, while we ran model inference on every available SAR image pair. Even with 4 GPUs, inference on 2 million plus image pairs took approximately 2 months (1400 compute hours). The computational challenges were driven by both data ingestion and running the neural network. Data was read in using Microsoft's Planetary Computer to identify the file locations in azure blob storage, and reading directly from the blob storage container for optimal data transfer speeds.

While running inference at a global scale over 10 years is challenging, because of the relatively small model size, inference on a pair of images is possible even on CPUs within minutes. This will make the model accessible to all, even those who do not have GPUs to run neural network models.

S2 Removal of False Positives

While water is typically identifiable in SAR imagery, one of the challenges with using SAR imagery is the potential for false positives. As mentioned in the Discussion section, there are multiple surface types that can mimic flooding under the right circumstances, even in a change detection model. In this section, we describe potential causes of false positives and how we address them with auxiliary datasets in our model post-processing.

S2.1 Sources of False Positives

Rough Terrain Areas with rough terrain, such as mountains and sand dunes, create shadowed regions with low backscatter amplitudes that can resemble water in SAR imagery. The shadowing effect caused by the terrain's elevation changes leads to decreased radar backscatter, making these areas appear dark, similar to smooth water surfaces.

Arid and semi-arid areas Arid regions like deserts often have low backscatter in SAR for two reasons. The first is that they often are smooth surfaces with little vegetation, meaning that any scattered light is scattered away from the satellite, similar to what happens with water. Second, the surface in many arid regions absorbs radar more readily than other surfaces, which further reduces the measured backscatter. Both of these processes can make these regions look similar to water in SAR.

Freeze-Thaw False Positives When the ground temperature is near or at the freezing point, the moisture in the soil can freeze or thaw. This significantly changes the dielectric properties of the surface. Since SAR signals are sensitive to changes in surface properties, such as roughness and dielectric constant (which itself is influenced by moisture content), these phase transitions can lead to signals that mimic those of a flooded surface. This is particularly true for cropland or other vegetated areas where the presence of moisture is common, and the freeze-thaw cycle can create a surface response in SAR imagery that resembles standing water.

S2.2 Post-processing

To counteract the false positives described above and artifacts in the underlying data, we apply multiple post processing steps after model inference.

Image edges Some Sentinel 1 SAR images, especially for the earlier years of Sentinel 1, have artifacts near the edges of images. These artifacts appear to have low (but non-zero) amplitude, and since these artifacts are not consistent, changes over time can manifest as flood detections in the model. To counter this, we apply simple heuristics to identify edges of images that have near uniform distributions of flood detections near the edge. If an edge is found to have a uniform distribution of points with 0.1° of the edge, we remove any flood detections in that range.

Soil Moisture We use soil moisture estimates derived from the AMSR-2 microwave satellite to remove false positives. We chose AMSR-2 data because we need soil moisture estimates that went back to Oct 2014, when the Sentinel-1 SAR data beings. Our approach to using soil moisture is to identify clear false positives: detections in mountains or in deserts, or for areas where we know it is not the typical flood season, and to use the maximum observed values of soil moisture as thresholds for false positives elsewhere.

We use the outputs of two different algorithms to estimate soil moisture. The first is the Land Parameter Retrieval Model

(LPRM), with soil moisture estimates available at 10 km resolution for three different frequency bands. The second is based on the Single Channel Algorithm (SCA) and is available at 25 km. We look at both the absolute values of soil moisture and compare to the historical trend using z-scores. We used two different algorithm outputs because we found that while the LPRM algorithm was the most useful in identifying potential false positives because of the higher resolution and more advanced algorithm, there were some scenarios in which the LPRM algorithm reported high soil moisture, typically in high elevation regions or near deserts where soil moisture is expected to be low. In some of these cases, results from the SCA algorithm were able to help filter out additional false positives.

The final heuristic we applied was to restrict flood detections based on both absolute soil moisture estimates and a soil moisture anomaly above 1 standard deviation. Specifically, the thresholds we chose were points with LPRM soil moisture above 0.2g/cm^3, SCA soil moisture above 0.01g/cm^3 and an LPRM z-score above 1.

We note that while we apply this, and all other filtering, to the global flood maps and the trends over time, we provide all raw flood detections from our model with the auxiliary data included so others can apply their own filters depending on their use case.

Digital Elevation Model We use the Copernicus GLO-30 Digital Elevation Model (DEM) to remove false positives due to rough terrain. We calculate the pixel-level slope from the DEM, and then apply a heuristic to remove any points with slope greater than 10° within 250m of the pixel. The choice of 10° is somewhat conservative, as it is very unlikely for water to accumulate on surfaces with slopes of 10° or more.

Land Surface Temperature We use the ERA5-Land surface temperature reanalysis to remove freeze-thaw false positives. For every date, we look at the minimum temperature, and if the minimum temperature is below freezing, we remove the flood detection.

Land Cover We use the ESA World Cover land use/land cover mapping, primarily to remove permanent water from the flood predictions. However, we also found that even after filtering out points via soil moisture thresholds and the DEM, we were getting false positives in desert areas. Given that these areas are extremely unlikely to have true flood events, we also filter out any flood detections in regions marked as ’Bare/Sparse Vegetation’ in ESA World Cover. We chose the ESA World Cover mappings for this instead of the ESRI land cover mappings used elsewhere in this paper for cropland identification because the ESA Land Cover marked more desert areas (for example, the majority of the Sahara) as ’Bare’, compared to ESRI land cover mappings, which often classified these areas as shrubland. Given that we wanted to remove anything in a desert like the Sahara, the ESA World Cover mapping was more aligned to our use case for the specific task of filtering out false positives in deserts.

S3 Flooding Trends Over Time

To estimate the change in flooding over time, we first aggregate the flood extent data by month, normalized by the number of available SAR observations. This results in a time series of mean flood extent per observation for each month from October 2014 to June 2024. It is important to normalize by the number of observations because while SAR observations are typically done on a consistent schedule with a return time period of 12 days per satellite, disruptions to this cadence can occur. For example, in late 2021 one of the two Sentinel-1 satellites went offline, greatly reducing the number of observations per month. Without normalizing by the number of observations, we could falsely conclude that flooding had decreased by nearly 50%.

We then fit a linear model to the time series, with dummy variables for the month of the year to account for seasonality and an index for the number of months since the beginning of the period. The primary coefficient of interest is the one associated with the index variable, as it indicates the average monthly increase in flood extent. We then convert this average monthly increase and its associated uncertainty to an average yearly increase.

While this approach provides a baseline for trend analysis, we identified several potential data issues that required additional consideration. For example, 2022 had anomalously high flood extent values, likely driven by major flood events such as the Pakistan flooding from June to October 2022. Additionally, most of the observations prior to June 2017 were conducted with a single polarization mode (VV, or Vertical-Vertical for sending and receiving radar signals vertically), rather than with both polarization modes (VV and VH, Vertical-Horizontal). This difference in polarization modes led to varying flood detection rates in the earlier data.

To correct for these different rates, we employed a simple pre-post causal inference analysis to estimate the difference in flood detection rates for single model vs dual mode detection. For each region, defined by latitude and longitude rounded to two decimal places (approximately 1 km by 1 km grids), we identified the last data point observed with just VV

polarization. This time is not the same for all parts of the world, which meant that we could not do a simple pre-post based on date across all regions. We then looked at the 12 months prior to that observation and the 12 months after and calculated the total flood extent and the number of observations. We then aggregated across all regions, using the last VV observation as the midpoint, to estimate the average flood extent per observation using only VV data and using VV and VH data combined. Supplementary Figure S6 shows the aggregate result for the pre-post time series. While noisy, there is a clear delta from the pre-period to the post-period. Given the significant change observed, we chose to use a pre-post approach instead of more complex causal inference techniques.

Since the pre-post analysis shows that the delta between the pre-period and post-period is noisy, we ran a bootstrap across the geographic regions to better understand the uncertainty in the estimate of the scale factor to apply to the VV-only observations. This allows us to quantify the uncertainty in our estimates of flood trends over time due to the correction on the VV data we have to do.

Ultimately, we ran multiple versions of the model to try to account for 2022 as a potential outlier and the different ways to account for the VV-only data:

- All data included
- 2022 excluded
- 2022 and pre-June 2017 data excluded
- 2022 excluded, lower limit on scale factor for early observations
- 2022 excluded, upper limit on scale factor for early observations

In the final results, we omitted the last scenario (upper limit on the scale factor) because it likely overestimates the trend in flooding over time. The results with the lower limit on the scale factor were roughly in line with the results where we exclude pre-June 2017 entirely, so for simplicity we only report the first three results in the main paper.

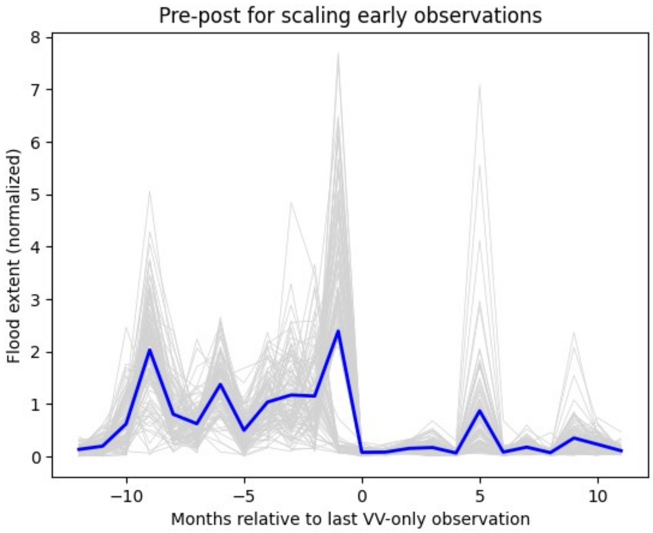

Supplementary Figure S6: Flood extent (normalized to pre period) measured for the earlier, VV-only observations (negative x values) and the later observations that include both VV and VH polarization channels (positive x values). We see a significant drop in flood detection rates when going from VV only observations to typical observations, even after removing false positives. The light gray lines represent 100 bootstrap iterations, illustrating the variability in this estimate.
